# A camel-derived MERS-CoV with a variant spike protein cleavage site and distinct fusion activation properties

**DOI:** 10.1038/emi.2016.125

**Published:** 2016-12-21

**Authors:** Jean Kaoru Millet, Monty E Goldstein, Rachael N Labitt, Hung-Lun Hsu, Susan Daniel, Gary R Whittaker

**Affiliations:** 1 Department of Microbiology and Immunology, College of Veterinary Medicine, Cornell University, Ithaca, NY 14853, USA; 2 Cornell Undergraduate Biology, Cornell University, Ithaca, NY 14853, USA; 3 Cornell DVM Program, Cornell University, Ithaca, NY 14853, USA; 4 School of Chemical and Biomolecular Engineering, Cornell University, Ithaca, NY 14853, USA; 5 Cornell Graduate Field of Chemical and Biomolecular Engineering, Cornell University, Ithaca, NY 14853, USA

**Keywords:** camel, coronavirus, furin, fusion activation, MERS, spike

## Abstract

Middle East respiratory syndrome coronavirus (MERS-CoV) continues to circulate in both humans and camels, and the origin and evolution of the virus remain unclear. Here we characterize the spike protein of a camel-derived MERS-CoV (NRCE-HKU205) identified in 2013, early in the MERS outbreak. NRCE-HKU205 spike protein has a variant cleavage motif with regard to the S2′ fusion activation site—notably, a novel substitution of isoleucine for the otherwise invariant serine at the critical P1′ cleavage site position. The substitutions resulted in a loss of furin-mediated cleavage, as shown by fluorogenic peptide cleavage and western blot assays. Cell–cell fusion and pseudotyped virus infectivity assays demonstrated that the S2′ substitutions decreased spike-mediated fusion and viral entry. However, cathepsin and trypsin-like protease activation were retained, albeit with much reduced efficiency compared with the prototypical EMC/2012 human strain. We show that NRCE-HKU205 has more limited fusion activation properties possibly resulting in more restricted viral tropism and may represent an intermediate in the complex pattern of MERS-CoV ecology and evolution.

## INTRODUCTION

Middle East respiratory syndrome coronavirus (MERS-CoV) is the most recently characterized human coronavirus, causing over 1800 reported infections to date and with a high case fatality rate above 35%. First reported in 2012, human MERS infections are still occurring, with a main focal point in the Arabian Peninsula, but with occasional imported cases to other countries.

MERS-CoV is classified as a clade c betacoronavirus, grouping with bat coronaviruses, such as BatCoV-HKU4 and BatCoV-HKU5. Its closest genetic relative is the bat coronavirus NeoCoV, infecting a South African bat species *Neoromicia capensis*.^1^ Although the origins of human MERS-CoV are still unclear, serological and sequencing studies have demonstrated that MERS-CoV infects dromedary camels in the Arabian Peninsula and in Africa. In particular, retrospective serological studies have shown that camels have been infected by MERS-CoV or MERS-CoV-like viruses well before 2012.^2^ Camel MERS-CoV infections are not associated with overt disease signs in animals, but are believed to be a source for human cases.

The coronavirus spike (S) protein is the main determinant of viral entry as it mediates both binding to the host cell receptor, dipeptidyl peptidase 4 (DPP4 or CD26)^3^ in the case of MERS-CoV, and fusion at cellular membranes. S is typically proteolytically processed for fusion by host cell proteases, a process that can occur at the S1/S2 site (located at the junction between the S1 receptor-binding and S2 fusion domains), and at the S2′ site (located upstream of the fusion peptide).^4^ Studies performed on the prototypical EMC/2012 S protein have shown that MERS-CoV represents an unusual coronavirus as it can be activated by a broad range of host cell proteases.^5, 6, 7, 8^ In particular, we and others have previously shown that a furin cleavage site present at S2′ is believed to add an extra ‘layer' of proteolytic activation enabling the virus to infect a wide variety of cells *in vitro,* possibly allowing the extra-pulmonary infection observed in MERS patients.^8, 9^

Since the first MERS-CoV genome was sequenced, many other human and camel-derived genome sequences have been published.^10, 11^ In this study, we examined the S protein of a divergent camel MERS-CoV isolate, NRCE-HKU205,^12^ for which the S protein sequence was previously shown to harbor several mutations, including two substitutions at the S2′ cleavage-activation site, A886S and S888I. We characterize the consequences of such substitutions on proteolytic cleavage and fusion activation.

## MATERIALS AND METHODS

### Cells and reagents

HEK-293 T (ATCC, Manassas, VA, USA), Huh-7 cells (Japan Health Science Research Resources Bank, Osaka, Japan), Vero-E6 cells (ATCC) and MRC-5 cells (ATCC) were grown at 37 °C 5% CO_2_ in DMEM (Corning, Corning, NY, USA) supplemented with 10% fetal bovine serum (ThermoFisher, Waltham, MA, USA), 10 mM HEPES (Corning), 100 IU/mL penicillin and 100 μg/mL streptomycin (Corning).

A mammalian codon-optimized gene encoding wild-type EMC/2012 MERS-CoV spike (EMC_wt_, GenBank: AFS88936.1) with a fused C-terminal C9-epitope tag was described previously,^8^ and subcloned in the pcDNA-3.1 vector. Mammalian codon-optimized wild-type NRCE-HKU205 spike (205_wt_, GenBank: AHL18090.1), and NRCE-HKU205 spike with S886A and I888S substitutions (205_EMC-S2__′_) containing C-terminal C9-epitope tag were synthesized (Biomatik, Wilmington, DE, USA) and subcloned in the pcDNA-3.1 vector. Site-directed mutagenesis (Agilent, Santa Clara, CA, USA) was performed to introduce A886S and S888I substitutions in the EMC/2012 S gene (EMC_205-S2__′_). The mutated gene sequence was verified by Sanger sequencing (Cornell Genomics Facility). pCMV-MLVgag-pol murine leukemia virus (MLV) packaging construct, pTG-Luc transfer vector encoding luciferase reporter and pCMV-Furin human furin-encoding vector were described previously.^13, 14^ The pCAGGS-VSV-G plasmid was used to generate positive control-pseudotyped particles.^8^

Fluorogenic peptides derived from MERS-CoV spike EMC/2012 and NRCE-HKU205 S2′ sites containing GSRSARSAIE and GSRSSRIAIE sequences, respectively, and harboring the (7- methoxycoumarin-4-yl)acetyl/2,4-dinitrophenyl (MCA/DNP) FRET pair were synthesized by Biomatik.

Recombinant human furin was purchased from New England Biolabs (Ipswich, MA, USA), recombinant cathepsin L was kindly provided by Dr Fang Li (University of Minnesota), and recombinant L-1-Tosylamide-2-phenylethyl chloromethyl ketone (TPCK)-treated trypsin was obtained from Sigma-Aldrich (St Louis, MO, USA). The furin inhibitor used in this study (dec-RVKR-CMK) was purchased from Tocris (Bristol, UK).

### Sequences, alignments and phylogenetic analyses

A phylogenetic tree of the spike protein from human and camel MERS-CoV as well as related bat coronaviruses was generated using the following full-length protein sequences provided by GenBank (ID in parenthesis): Jordan-N3/2012 (AGH58717.1), EMC/2012 (AFS88936.1), Riyadh-3/2013 (AGV08390.1), England-1/2012 (AFY13307.1), Jeddah-Camel-1/2013 (AHE78097.1), Taif-1/2013 (AHI48594.1), Wadi-Ad-Dawasir-1/2013 (AHI48550.1), Riyadh/Ry179/2015 (ALA49902.1), Jeddah/Jd1(b)/2015 (ALA49715.1), Jeddah/D90/2014 (ALA49561.1), KOR/KNIH/002-05/2015 (AKL59401.1), FL/USA-2-Saudi-Arabia/2014 (AHZ64057.1), Qatar-2/2014 (AHX71946.1), Hafr-Al-Batin-1/2013 (AGV08455.1), Al-Hasa-1/2013 (AGN70962.1), KFU-HKU-1/2013 (AHX00731.1), FRA/UAE/2013 (AHB33326.1), NRCE-HKU205/2013 (AHL18090.1), NeoCoV/2011 (AGY29650.2), BetaCoV-SC2013/2013 (AHY61337.1), HKU5-1/LMH03f/2007 (ABN10875.1), BtCoV/133/2005 (ABG47052.1), HKU4-1/B04f/2007 (ABN10839.1). The tree was generated using the Neighbor-Joining (NJ) clustering algorithm performed with the Geneious 10 software package (Biomatters, Auckland, New Zealand) and was then formatted using FigTree software (http://tree.bio.ed.ac.uk/software/figtree/).

Sequence alignment of the spike S2′ site of representatives of each coronavirus genus was performed by ClustalW alignment (Geneious) with gaps removed. The following sequences were used, along with their GenBank ID in parenthesis: BatCoV-HKU10 (AFU92104.1), PEDV-CV777 (AAK38656.1), FCoV-RM (ACT10854.1), CCoV-Elmo/02 (AAP72149.1), HCoV-NL63 (AGT51331.1), HCoV-229E (AAK32188.1), FCoV-1683 (AFH58021.1), CCoV-1-71 (AAV65515.1), TGEV-Miller M6 (ABG89301.1), TGEV-Purdue (CAB91145.1), MHV-A59 (ACO72884.1), MHV JHM (CAA28484.1), BCoV-Quebec (BAA00557.1), HCoV-OC43 (AAX84791.1), HCoV-HKU1 (AAT98580.1), SARS-CoV (AAP13441.1), MERS-CoV EMC/2012 (AFS88936.1), MERS-CoV NRCE-HKU205 (AHL18090.1), NeoCoV (AGY29650.2), BatCoV-HKU4 (ABN10839.1), BatCoV-133 (ABG47052.1), BatCoV-SC2013 (AHY61337.1), BatCoV-HKU5 (ABN10875.1), IBV-Beaudette (AAA70235.1), IBV-M41 (AAW33786.1), IBV-Cal99 (AAS00080.1), TCoV-MG10 (ABW81427.1), BeCoV-SW1 (ABW87820.1), BdCoV-HKU22 (AHB63481.1), BuCoV-HKU11 (ACJ12044.1), ThCoV-HKU12 (ACJ12053.1), MuCoV-HKU13 (ACJ12062.1). Similarly, a protein alignment of the S2′ sites derived from 386 camel and human MERS-CoV was performed (Geneious). The GenBank accession number for each sequence is provided within Supplementary Figure S1.

MERS-CoV EMC/2012 (AFS88936.1) and MERS-CoV NRCE-HKU205 (AHL18090.1) S2′ sites were scored for predicted furin cleavability using PiTou 2.0 software.^15^ A positive score indicates prediction of furin cleavability while a negative score indicates a sequence not predicted to be cleaved by furin. Prediction of furin cleavage based on comparison of amino-acid composition of known furin substrates found in the MEROPS protease database was also performed.^16^

### Fluorogenic peptide assays

For each fluorogenic peptide, a reaction was performed in a 100 μL volume with buffer composed of 100 mM Hepes, 0.5% Triton X-100, 1 mM CaCl_2_ and 1 mM 2-mercaptoethanol pH 7.5 for furin (diluted to 10 U/mL), or PBS for trypsin (diluted to 50 ng/μL), or 25 mM MES pH 5.0 in the case of cathepsin L (diluted to 1 μg/mL), with the peptide diluted to 50 μM. Reactions were performed at 30 °C in triplicates, and fluorescence emission was measured every minute for 45 min using a SpectraMax fluorometer (Molecular Devices, Sunnyvale, CA, USA), with *λ*_ex_ 330 nm and *λ*_em_ 390 nm wavelengths setting, enabling tracking of fluorescence intensity over time and calculation of *V*_max_ of reactions.

### Pseudotyped virus production

MLV-based MERS-CoV S-pseudotyped particles harboring EMC_wt_, EMC_205-S2__′_, 205_wt_ and 205_EMC-S2__′_ S proteins, or the vesicular stomatitis virus glycoprotein (VSV-G), or without envelope glycoproteins—‘bald' particles (Δenv), were generated as previously described.^3^ Briefly, HEK-293 T cells were transfected with MERS-CoV S-encoding plasmids (EMC_wt_, EMC_205-S2__′_, 205_wt_ and 205_EMC-S2__′_ S particles), VSV-G-encoding plasmid (VSV-G particles), or an empty vector (Δenv particles), along with pCMV-MLVgag-pol packaging construct and the MLV transfer vector encoding luciferase reporter, using Lipofectamine 2000 transfection reagent (Life Technologies, Carlsbad, CA, USA). The cells were incubated at 37 °C 5% CO_2_ for 48 h, and supernatants were harvested, filtered through 0.45-μm membranes and stored at −80 °C until used for particle concentration determination, infection or western blot assays.

### Pseudotyped viral particle concentration quantification

Quantification of the concentration of pseudotyped viral particles produced was performed using nanoparticle tracking analysis (NTA) methodology with a Nanosight NS300 device and NTA 3.0 software (Malvern, Malvern, UK), located at Cornell University's Nanobiotechnology Center (NBTC). Pseudotyped particle concentration was determined on a particle-by-particle basis by analysis of laser illumination scattering by particles using microscopy video recording analysis. Three recordings (60 s each) were performed on each sample at a constant temperature of 22.5 °C, and the average of concentration measurements was calculated for particles with an average size of ~120 nm. The device was cleaned thoroughly before and after each use.

### Pseudotyped virus western blot assays

For analysis of S cleavage, pseudotyped particles were ultracentrifuged at 42 000 rpm for 2 h at 4 °C, using a TLA-55 rotor with an Optima-MAX-E centrifuge (Beckman-Coulter, Brea, CA, USA). Viral pellets were resuspended in a buffer containing 100 mM Hepes, 0.5% Triton X-100, 1 mM CaCl_2_ and 1 mM 2-mercaptoethanol. For exogenous furin treatment of pseudovirions, 6 U of recombinant human furin were added (New England Biolabs) and the proteolytic reactions were performed at 37 °C. Lithium dodecyl sulfate loading buffer and DTT were added to samples, which were then heated at 95 °C for 5 min. Protein samples were separated on NOVEX Bis-Tris gels (Life Technologies) and transferred on polyvinylidene fluoride membranes (Sigma-Aldrich), with MERS-CoV S detection performed using an anti-C9 tag antibody (1D4, Abcam, Cambridge, MA, USA) and MLV capsid detection performed using a mouse monoclonal anti-MLV capsid p30 antibody (4B2, Abcam). Western blot signal detection was done by adding reagents of the ECL kit from Pierce (Rockford, IL, USA) and image acquisitions were performed using an LAS-3000 imager (FujiFilm, Tokyo, Japan).

### MERS-CoV S-induced cell–cell fusion assays

A total of 1.25 × 10^5^ Huh-7 cells were seeded in wells of microscopy slide chambers (Ibidi, Martinsried, Germany) and incubated at 37 °C 5% CO_2_ for 24 h. The cells were transfected with plasmids encoding MERS-CoV EMC_wt_, EMC_205-S2__′_, 205_wt_ and 205_EMC-S2__′_ S proteins with or without cotransfection of a human furin-encoding plasmid. The transfected cells were left untreated, treated with exogenous furin (25 U/mL), or treated with 75 μM furin inhibitor for 24 h. Transfected cells were fixed, permeabilized and immunolabeled for S expression using primary rabbit anti-MERS S polyclonal antibody (40069-RP02, SinoBiological, Beijing, China) and secondary AlexaFluor-568-conjugated goat anti-rabbit IgG antibodies (Invitrogen, CA, USA). Nuclei were stained with DAPI. Immunofluorescence microscopy analysis was performed with a Zeiss Axiovert 200M microscope (Jena, Germany), using a × 20 objective for image acquisition. To quantify the extent of syncytia formation, for each condition, 10 syncytia were analyzed for the number of nuclei they contained with a × 10 objective, and the average number of nuclei/syncytium was calculated.

### Pseudotyped virus infectivity assays

A total of 2.5 × 10^5^ Huh-7, Vero-E6 and MRC-5 cells were seeded in 24-well plates and incubated at 37 °C for 24 h. The cells were washed with PBS, and 200 μL pseudotyped particles, corresponding to an average of 1.8 × 10^7^ particles for all pseudovirion types as determined by nanoparticle tracking analysis (Supplementary Figure 2A), were added to cells and incubated at 37 °C for 2 h. Complete medium was then added and cells were incubated at 37 °C for 72 h, after which luciferase activity was measured using Luciferase Assay Kit (Promega, Madison, WI, USA), and luminometer readings performed with a GloMax 20/20 system (Promega).

### Statistical analyses

Quantitative data were analyzed and plotted using GraphPad Prism 7 (GraphPad Software, La Jolla, CA, USA). Statistical significance analyses were performed using two-tailed Student's *t-*test. To describe *P-*value significance, the following convention was used: not significant (NS), *P*>0.05; significant (*), *P*≤0.05; highly significant (**), *P*≤0.01; and very highly significant (***), *P*≤0.001.

## RESULTS

**Figure 1:**
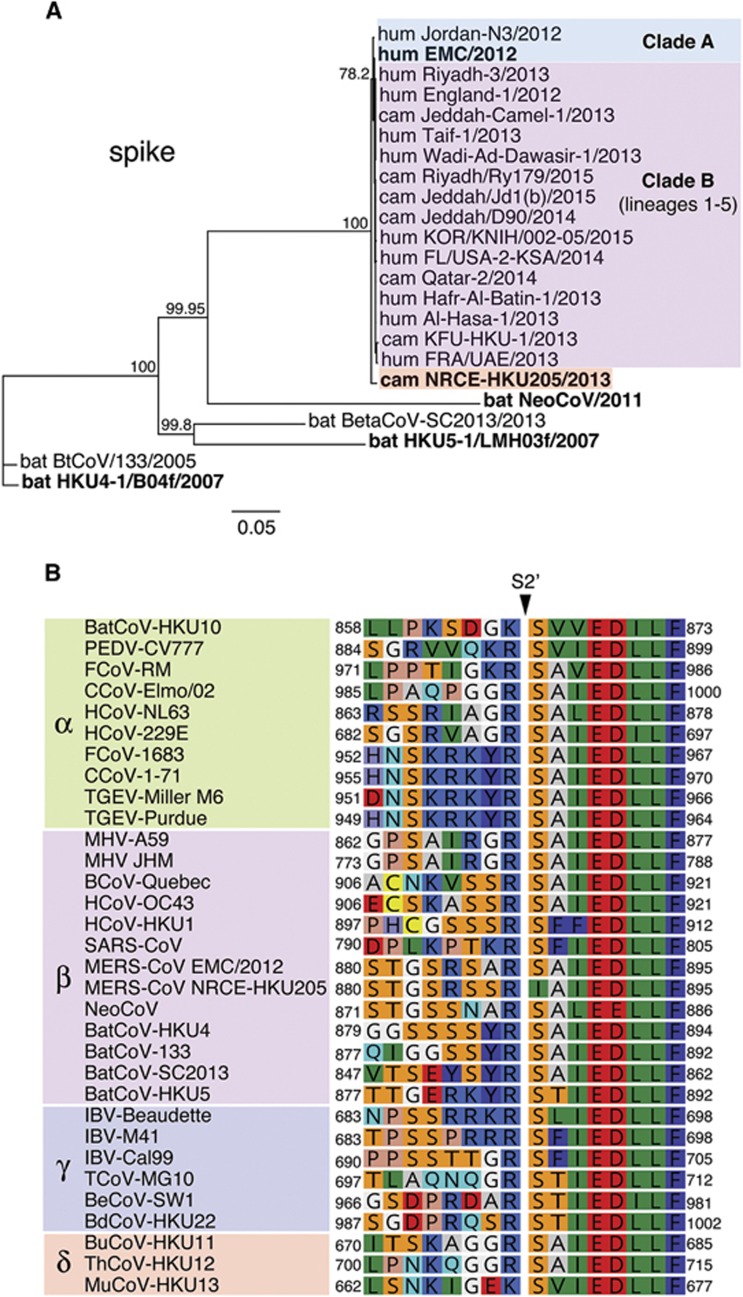
Sequence analysis of the spike protein of camel MERS-CoV NRCE-HKU205. (**A**) Phylogenetic analysis of full-length spike protein of human and camel strains of MERS-CoV and related bat coronaviruses. The phylogenetic tree was generated using the Neighbor-Joining method (bootstrap resampling, 2000 replicates) with Geneious software from spike protein sequence alignment of representative MERS-CoV strains along with closely related bat betacoronaviruses retrieved from GenBank (accession numbers in methods section). Numbers near nodes represent percent consensus support. Scale bar represents estimated number of substitutions per site. camel, cam; human, hum. (**B**) Protein sequence alignment of coronavirus spike S2′ cleavage sites. Protein sequences of the S2′ cleavage site of representative coronaviruses from all four genera (accession numbers in methods section) were aligned using the ClustalW alignment method in Geneious software. Numbers indicate residue position within individual spike proteins.

On the basis of analyses conducted on the prototypical EMC/2012 strain, we previously demonstrated that the MERS-CoV spike contained furin cleavage motifs at both S1/S2 and S2′ cleavage sites,^8^ which are consistently observed in the many other MERS-CoV genome sequences subsequently derived from both human and camels.^10, 11^ However, Chu and colleagues have reported the nearly complete genome of a camel MERS-CoV isolate, NRCE-HKU205 (referred to as HKU205) from an infected animal located at a Cairo slaughter house, but imported from Sudan or Ethiopia.^12^ HKU205 has a spike protein containing 12 amino-acid substitutions and a deletion of 1 amino-acid, compared with EMC/2012. Intriguingly, while its S1/S2 site is identical to the one found in EMC/2012 spike, the S2′ cleavage site was found to contain two substitutions: S886A and S888I. To contextualize this finding with more recent sequencing data and to identify other possible variants, we performed comprehensive sequence alignments of MERS-CoV spike S2′ sequences available on GenBank (Supplementary Figure S1). We identify HKU205 S2′ as the only reported variant sequence in an otherwise invariant site for the 386 sequences analyzed. Phylogenetic analysis of the spike protein sequence reveals that HKU205 is divergent and forms a basal sister relationship with clade A and B MERS-CoV sequences (Figure 1A). The sequence for HKU205 S2′ was analyzed and compared with those of representative coronaviruses from all four genera (Figure 1B). While S2′ cleavage motifs show a high degree of variability amongst coronaviruses, the sequence immediately downstream of it, recognized as being the coronavirus fusion peptide,^9, 17, 18, 19^ is extremely well conserved. However, HKU205 is the only known coronavirus that contains an isoleucine instead of a serine at the residue position immediately following the cleavage site, corresponding to the P1′ position of the furin cleavage motif.

**Figure 2:**
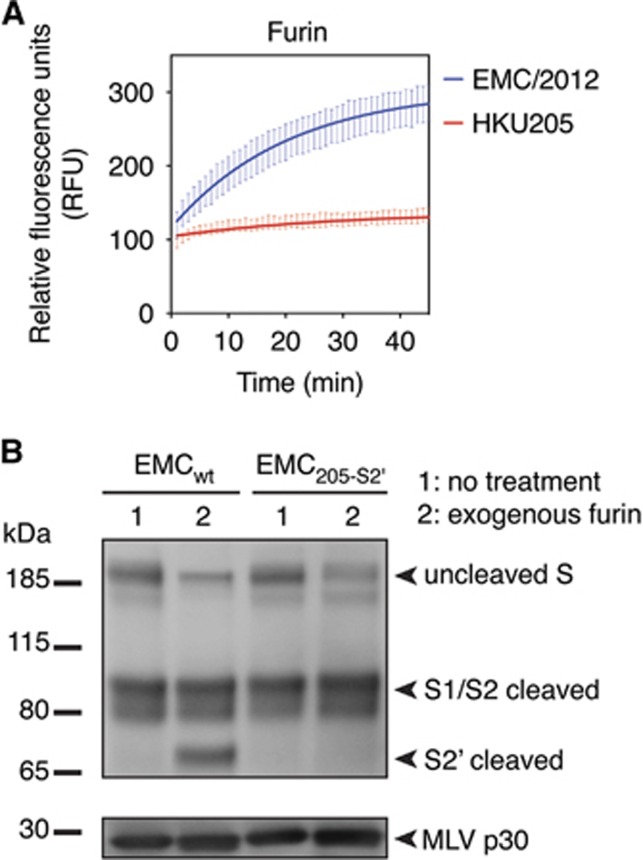
Effect of HKU205 S2′ substitutions on furin proteolytic cleavage. (**A**) Furin cleavage assay of fluorogenic peptides. Fluorogenic peptide mimetics of the S2′ spike cleavage sites of MERS-CoV strains EMC/2012 (blue line) and HKU205 (red line) were incubated with recombinant furin and the increase in fluorescence due to proteolytic processing was measured using a fluorometer. Assay performed in triplicates with results representing averages from three independent experiments of relative fluorescence units over time (*n*=3). Error bars indicate SD. (**B**) Analysis of the consequences of HKU205 S2′ substitutions on furin proteolytic processing products of MERS-CoV S by western blot. Murine leukemia virus (MLV) pseudovirions bearing EMC/2012 S protein with either a wild-type (EMC_wt_) or with a HKU205 substituted S2′ cleavage site (EMC_205-S2__′_) were either untreated or treated with exogenous furin. The samples were analyzed by western blot to compare proteolytic cleavage of S proteins. The MLV capsid protein p30 was used as loading control.

The unusual features of HKU205 S2′ among MERS-CoV isolates and coronaviruses in general prompted us to investigate further the implications of such variation on spike protein activation. Despite still containing two paired basic arginine residues, which are typically found in furin cleavage sites, the HKU205 S2′ sequence is not predicted to be cleaved by furin according to PiTou 2.0 cleavability scoring algorithm (Table 1).^15, 20^ Furthermore, the MEROPS protease database indicates that of the 208 known furin-cleaved substrates, none contain an isoleucine at the position immediately after the scissile bond (P1′).^16^ To validate these predictions, we performed fluorogenic peptide cleavage assays and demonstrate that while furin was able to proteolytically process EMC/2012 S2′-derived peptide (*V*_max_=7.3±0.2 relative fluorescence units (RFU)/min), the HKU205 S2′ peptide could not be cleaved by the protease (*V*_max_=0.6±0.3 RFU/min) (Figure 2A and Table 1). To specifically assess the effect of introducing the S2′ mutations, we generated by site-directed mutagenesis a full-length EMC/2012 spike protein harboring the S886A and S888I substitutions (EMC_205-S2__′_ S) and performed a western blot analysis on MLV-based pseudovirions harboring either wild-type (EMC_wt_) or mutated (EMC_205-S2__′_) S proteins (Figure 2B). In the non-treated condition (lane 1) EMC_wt_ S migrated with the same band pattern as shown previously,^8^ with full-length (uncleaved) protein and S1/S2 cleavage product due to furin processing during S protein maturation in producer cells. Treatment of EMC_wt_ S with recombinant furin (lane 2) generates a new band (above 65 kDa marker) corresponding to proteolytic processing at the S2′ site. Although the EMC_205-S2__′_ S band pattern is identical to the one observed for EMC_wt_ in the non-treated condition (lane 1), mutating the S2′ site abrogates furin cleavage, as no S2′ band was observed for EMC_205-S2__′_ S (lane 2). These results confirm that the HKU205 substitutions within the S2′ cleavage site block furin cleavability.

**Figure 3:**
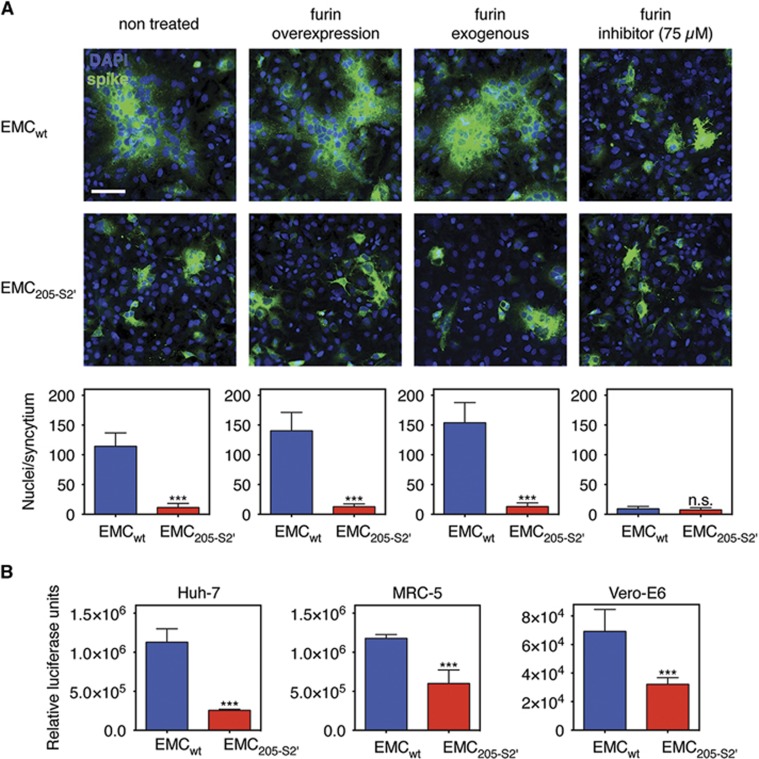
Impact of HKU205 S2′ substitutions on fusion-activation and host cell entry mediated by MERS-CoV S. (**A**) Assessment of the effect of HKU205 S2′ substitutions on MERS-CoV S fusogenicity. Huh-7 cells were transfected to express MERS-CoV EMC/2012 S with either wild-type (EMC_wt_) or HKU205 substituted S2′ site (EMC_205-S2__′_). The cells were either untreated, co-transfected with a furin-encoding plasmid (furin overexpression), treated with exogenous furin (furin exogenous) or treated with 75 μM of the furin inhibitor dec-RVKR-CMK (furin inhibitor). The cells were then processed for immunofluorescence with labeling of S protein (false colored green) and nuclei (DAPI, blue). Scale bar represents 100 μm. Quantitative microscopy analysis was undertaken to assess the average sizes of syncytia induced by S expression. Ten syncytia were analyzed for each condition and data are the averages of nuclei/syncytium from three independent experiments (*n*=3). (**B**) Effect of HKU205 S2′ substitutions on MERS-CoV S-mediated host cell entry. MLV-pseudotyped viruses harboring EMC/2012 S protein with either a wild-type (EMC_wt_) or with a HKU205 substituted S2′ cleavage site (EMC_205-S2__′_) and containing a luciferase reporter gene were used to infect human Huh-7 (liver) and MRC-5 (lung) cells along with simian Vero-E6 (kidney) cells. 72 h post infection, cells were assayed for luciferase activity using a luminometer. Assays performed in triplicates and results are averages of relative luciferase units from two independent experiments (*n*=2). For both **A** and **B**, error bars indicate SD and statistical significance analyses were performed using two-tailed Student's *t*-test. Not significant (NS), *P*>0.05; very highly significant, ***P≤0.001.

We examined the functional consequence of the abrogation of furin processing in the HKU205 S2′ spike cleavage site by performing a cell–cell fusion assay in transfected Huh-7 cells expressing either EMC_wt_ or EMC_205-S2__′_ spike proteins (Figure 3A). Expression of EMC_wt_ S is accompanied by the formation of spontaneous large syncytia (non-treated condition). Furin overexpression or treatment with exogenous recombinant furin increase the average size of syncytia (from an average of ~114 nuclei to ~140–150 nuclei). Furthermore, inhibition of furin activity by treatment with 75 μM of the furin inhibitor dec-RVKR-CMK abrogated syncytia formation. In contrast, expression of EMC_205-S2__′_ S was not accompanied by syncytia formation in all conditions tested, including when furin is overexpressed or present exogenously. These data confirm that the HKU205 S2′ mutations abrogate furin-mediated fusion activation. Next, the luciferase gene reporter-containing MLV pseudovirions harboring either EMC_wt_ or EMC_205-S2__′_ S were further analyzed. Quantification by nanoparticle tracking analysis of the concentration of both types of pseudovirions shows that the introduction of the 205-S2′ mutation did not significantly alter particle concentration produced compared with EMC_wt_ S-pseudotyped particles (Supplementary Figure 2A). EMC_wt_ or EMC_205-S2__′_ S-pseudotyped particles were used to infect Huh-7, Vero-E6 and MRC-5 cells (Figure 3B), along with control-pseudotyped particles: ‘bald' or no envelope particles (Δenv), and vesicular stomatitis virus glycoprotein-pseudotyped particles or VSV-G (Supplementary Figure 2B). For the three cell lines, Δenv particle infectivity was close to background levels observed in the non-infected (NI) control conditions (~10^2^ range of relative luciferase units or RLU). When positive control VSV-G particles were assayed, infectivity measurements rose to ~10^6^ (Vero-E6) and ~10^7^ (Huh-7 and MRC-5) RLU levels, confirming that this system allows measurement of heterologous viral envelope protein-mediated entry and infectivity. For the MERS S-pseudotyped particles, in all cell lines tested, a significant drop in infectivity was observed for EMC_205-S2__′_ S-pseudotyped particles compared with EMC_wt_ S-pseudotyped particles, but the most pronounced effect was in Huh-7 cells (4.4-fold decrease) that naturally express high levels of furin.^8^ Since there were no significant differences in viral concentrations between EMC_wt_ and EMC_205-S2__′_ S-pseudovirions (Supplementary Figure 2A), we attribute the drop in infectivity to the mutation of the S2′ site in the EMC_205-S2__′_ S protein. These results suggest that while HKU205 S2′ can still be activated by other host cell proteases, the loss of furin cleavability decreases its fusogenic activity.

**Figure 4:**
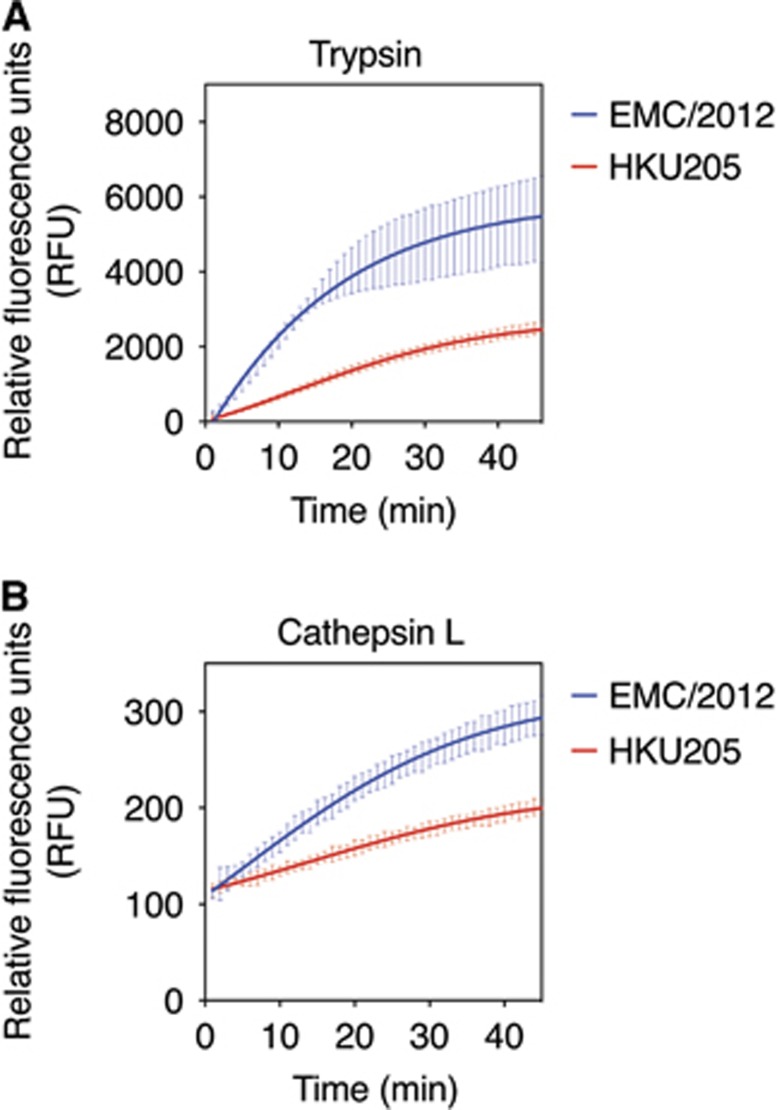
Analysis of proteolytic cleavage of HKU205 S2′ site by trypsin and cathepsin L. (**A**) Trypsin cleavage assay of fluorogenic peptides. Fluorogenic peptide mimetics of the S2′ spike cleavage sites of MERS-CoV strains EMC/2012 (blue line) and HKU205 (red line) were incubated with TPCK-treated trypsin and the increase in fluorescence due to proteolytic processing was measured using a fluorometer. (**B**) Cathepsin L cleavage assay of fluorogenic peptides. Assay performed as in **A** with cathepsin L used instead of trypsin. Both trypsin and cathepsin L cleavage assays were performed in triplicates with results representing averages of relative fluorescence units over time from three independent experiments (*n*=3). Error bars indicate SD.

Previous studies have shown that MERS-CoV S could be proteolytically activated by various host cell proteases, including furin, cathepsins and trypsin-like proteases.^5, 6, 7, 8^ To test whether HKU205 S2′ could still be cleaved by proteases other than furin, we performed fluorogenic peptide cleavage assays of both EMC/2012 and HKU205 S2′ peptides with either trypsin or cathepsin L (Figure 4 and Table 2). Trypsin cleaved both EMC/2012 and HKU205 S2′ peptides, with the EMC/2012 peptide still retaining a higher cleavage rate (EMC/2012 *V*_max_=220.5±3.4 RFU/min, HKU205 *V*_max_=67.6±1.3 RFU/min). Cathepsin L cleavage was detected for the EMC/2012 peptide (*V*_max_=5.8±0.2 RFU/min), and to a much lesser extent for HKU205 S2′ (*V*_max_=2.2±0.1 RFU/min). *V*_max_ data for the proteases tested in this study are summarized in Table 2, and along with western blot data, suggest that HKU205 S has a more restricted range of activating proteases than EMC/2012, being unable to utilize furin and likely more dependent on trypsin-like proteases.

**Figure 5:**
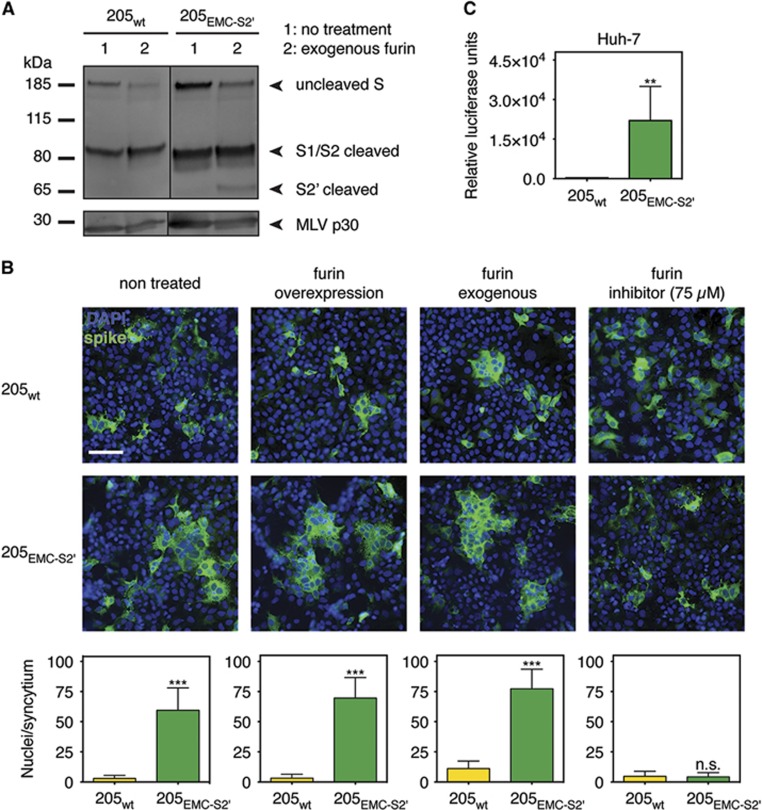
Effect of introducing the EMC/2012 S2′ furin cleavage site in HKU205 S. (**A**) Analysis of furin cleavage products. MLV-pseudotyped viruses bearing HKU205 S protein with either a wild type (205_wt_) or with a EMC/2012 substituted S2′ cleavage site (205_EMC-S2__′_) were either untreated or treated with exogenous furin. The samples were analyzed by western blot to compare proteolytic cleavage products of S proteins. The MLV capsid protein p30 was used as loading control. (**B**) Impact of the introduction of the EMC/2012 S2′ furin cleavage site on HKU205 S-mediated fusion. Huh-7 cells were transfected to express MERS-CoV HKU205 S with either wild type (205_wt_) or EMC/2012 substituted S2′ site (205_EMC-S2__′_). The cells were either untreated, co-transfected with a furin-encoding plasmid (furin overexpression), treated with exogenous furin (furin exogenous), or treated with 75 μM of the furin inhibitor dec-RVKR-CMK (furin inhibitor). The cells were then processed for immunofluorescence with labeling of S protein (false colored green) and nuclei (DAPI, blue). Scale bar represents 100 μm. Quantitative microscopy analysis was undertaken to assess the average sizes of syncytia induced by S expression. Ten syncytia were analyzed for each condition and data are the averages of nuclei/syncytium from three independent experiments (*n*=3). (**C**) Effect of introducing the EMC/2012 S2′ furin cleavage site on HKU205 S-mediated host cell entry. MLV-pseudotyped viruses harboring HKU205 S protein with either a wild type (205_wt_) or with a EMC/2012 S2′ furin cleavage site (205_EMC-S2__′_) and containing a luciferase reporter gene were used to infect human Huh-7 (liver) cells. Seventy-two hours post infection, cells were assayed for luciferase activity using a luminometer. Assays performed in triplicates and results are averages of relative luciferase units from two independent experiments (*n*=2). For both **B** and **C**, error bars indicate SD and statistical significance analyses were performed using two-tailed Student's *t*-test. Not significant (NS), *P*>0.05; highly significant, **P≤0.01; very highly significant, ***P≤0.001.

As furin proteolytic processing of MERS-CoV S S2′ was suggested to add an extra layer of proteolytic activation, we analyzed the effect of introducing the EMC/2012 S2′ furin site within full-length HKU205 S (Figure 5 and Supplementary Figure 2). Wild-type HKU205 S (205_wt_) protein, and a HKU205 S variant containing the EMC/2012 S2′ cleavage site (205_EMC-S2__′_) were synthesized, incorporated into MLV pseudovirion and analyzed by western blot (Figure 5A). Although 205_wt_ S is not cleaved at S2′ upon furin treatment (205_wt_ lane 2), the introduction of EMC/2012 S2′ in the HKU205 S background allows for detection of the S2′ cleaved product. We next performed a cell–cell fusion assay on Huh-7 cells expressing either 205_wt_ and 205_EMC-S2__′_ S (Figure 5B). 205_wt_ S displayed low fusogenicity in all conditions tested, a situation reminiscent of the EMC_205-S2__′_ S condition in Figure 3A. The introduction of the EMC/2012 S2′ site in the HKU205 S background allowed for the formation of spontaneous syncytia upon expression (average of ~60 nuclei/syncytium), which increased in average size when furin was overexpressed or when exogenous furin was present (~70 and ~77 nuclei/syncytium, respectively). Furin inhibitor treatment abrogated the formation of 205_EMC-S2__′_ S-induced syncytia. These results confirm that switching the HKU205 S2′ site to the EMC/2012 S2′ site in the HKU205 S background allows for enhanced furin-mediated fusogenicity of the fusion protein. The 205_wt_ and 205_EMC-S2__′_ S-pseudotyped particles were subsequently assayed for particle concentration by nanoparticle tracking analysis (Supplementary Figure 2A). These measurements showed that there were no statistically significant differences in concentration of particles produced between 205_wt_ and 205_EMC-S2__′_ S-pseudotyped particles and between these particles and the EMC_wt_ and EMC_205-S2__′_ S-pseudotyped particles. The pseudovirions were then assayed for infection in Huh-7 cells (Figure 5C). 205_wt_ S-pseudotyped particles displayed low infectivity (2.65 × 10^2^ RLU) close to NI and Δenv conditions (Supplementary Figure 2B). However, a significant ~2 log_10_ increase of infectivity is observed when EMC/2012 S2′ furin cleavage site is present (2.21 × 10^4^ RLU), confirming that the furin-cleaved S2′ site enhances fusion activation and infectivity. These analyses confirm that the narrow spectrum of activating proteases capable of processing HKU205 S2′ decreases viral infectivity and suggest that this strain may have a reduced cell and host tropism range.

## DISCUSSION

The human MERS epidemic remains a public health concern as it is associated with a high mortality rate, continues to affect populations in the Middle East with no vaccines or therapeutics available.^21^ Much like SARS-CoV, MERS-CoV exemplifies the propensity of coronaviruses to cross species barriers.^22, 23^ Since the beginning of the epidemic, hundreds of viral genomes from both human and camel hosts have been sequenced. Although comparative genetic characterizations have been conducted,^10, 11, 12, 24, 25^ few studies, if any, have analyzed the functional consequences of amino-acid variations identified by comparing MERS-CoV genomes.

In this work, we have studied the biological impact of substitutions found in the S2′ fusion activation site of the S protein of a divergent camel-derived MERS-CoV isolate, HKU205.^12^ We have found that the A886S and S888I substitutions are unique to HKU205 among sequenced MERS-CoV strains. In fact, comparative sequence analysis with major representatives of all coronavirus genera shows that the serine to isoleucine substitution at position 888, corresponding to the P1′ position in the furin cleavage site, is unique to HKU205. Intriguingly, the protein alignments we performed revealed that the human coronavirus HCoV-NL63 (AGT51331.1) contained an identical sequence (_863_RSSRIA_868_) to the one found in HKU205, but located a few residues upstream of S2′. Although HCoV-NL63 has been suggested to have a furin cleavage motif at S2′,^9^ it is possible that this cleavage motif is biochemically ‘blocked' for furin cleavage in a similar manner to HKU205.

With the exception of HKU205, we also noted an overall high level of conservation in the S protein sequence of camel and human MERS-CoV strains. These findings highlight the distinct nature of HKU205. Overall, we show that the variation found in HKU205 S2′ confers a decreased range of proteases for fusion activation. These findings suggest that HKU205 has a limited ability to cross the species barrier, and that the furin site found in most MERS-CoV samples may have been an important contributing factor for zoonotic transmission of the virus.

In this study we have focused on the substitutions found in the S2′ site of HKU205. However, there are ten other substitutions and 1 amino-acid deletion present in the spike protein of HKU205.^12^ The cell–cell fusion assays we performed have shown that introducing the EMC/2012 S2′ site in the HKU205 S background was accompanied by significant enhancement of fusogenicity, but to levels that did not reach the ones observed for wild-type EMC/2012 S. Likewise, the pseudotyped particle infectivity assays have shown that wild-type HKU205 S-pseudovirions have very little infectivity, while substituting its S2′ site with the furin site found in EMC/2012 significantly increased infectivity in Huh-7 cells, but did not restore it to the levels observed for wild-type EMC/2012 S-pseudovirions. Further investigation is required to understand the biological consequence of the other reported variations found in HKU205 S, particularly the substitutions in the S1 receptor-binding domain. In addition, because attempts to culture HKU205 virus have so far been unsuccessful,^12^ we used a mammalian codon-optimized gene expression strategy, which allowed us to robustly express and analyze all MERS-CoV S variant proteins. However, the expression levels we have used here are likely to be different from the ones occurring during viral infection. We have previously shown that for the EMC/2012 MERS-CoV strain, furin-mediated cleavage was similar for S proteins on native virions and on MLV-pseudotyped particles generated by expression of mammalian codon-optimized S gene.^8^ If HKU205 virus cultures become available, it would be interesting to carry out a comparative analysis of S expression, cleavage and fusogenicity on native virions and in infected cells.

Although rare, the fact that variations can occur in the MERS-CoV S2′ site is of concern, because it is known for other pathogenic viruses that alterations in the cleavage-activation mechanism can have a profound impact on tropism and pathogenicity. This is well documented for the hemagglutinin protein of highly pathogenic avian influenza strains.^26^ In the case of coronaviruses, the cleavage site changes in HKU205 are reminiscent of the mutations selected in the spike S1/S2 and S2′ cleavage sites of feline coronaviruses that are macrophage-tropic and the cause of lethal peritonitis in cats.^27, 28^ For MERS-CoV, we have shown previously that the S2′ furin cleavage site is not optimal,^8^ and so may be only a few mutational steps away from acquiring a more efficiently processed polybasic furin site. The implications of this for viral pathogenesis remain unknown.

Our work highlights the importance of surveillance of circulating MERS-CoV in both camels and humans followed by functional analyses of the biological impact of variations uncovered. Further sequencing and functional studies should be conducted to characterize the frequency and effects of other variations.

## Figures and Tables

**Table 1:** Comparison of predicted and measured furin-mediated proteolytic processing of EMC/2012 and HKU205 MERS-CoV S2′ sites

**Spike protein**	**Sequence**	**PiTou score**	**MEROPS**	**Furin *V*_max_**
EMC/2012	TGS**R**SA**R**SAIEDL	9.70	+	7.3±0.7
NRCE-HKU205	TGS**R**SS**R**IAIEDL	−32.49	−	0.6±0.3

Furin cleavage prediction scores were calculated using PiTou 2.0 software. A positive score indicates prediction of furin cleavage, while a negative score indicates that furin cleavage is not predicted. The sequences were also compared with known furin cleavage substrates found in the MEROPS database. A + sign indicates that residues in the sequence fit with known furin substrates, while a − sign indicates that at least one residue in the cleavage site is not compatible with furin cleavage. The average values of the rate of furin proteolytic processing of fluorogenic peptides (*V*_max_) are indicated and expressed as RFU per minute.

**Table 2:** Summary of rates of proteolytic cleavage (*V*_max_) of EMC/2012 and HKU205 MERS-CoV S2′ sites

**Spike protein**	**Control *V*_max_**	**Trypsin *V*_max_**	**Cathepsin L *V*_max_**	**Furin *V*_max_**
EMC/2012	0.3±0.2	220.5±3.4	5.8±0.2	7.3±0.7
NRCE-HKU205	−0.1±0.2	67.6±1.3	2.2±0.1	0.6±0.3

The average values of the rate (*V*_max_) of trypsin, cathepsin L and furin proteolytic processing of fluorogenic peptides are summarized and expressed as RFU per minute. Control conditions correspond to assaying the fluorogenic peptides without protease.
