# Supplementary material for: A camel-derived MERS-CoV with a variant spike protein cleavage site and distinct fusion activation properties
Source: Emerg Microbes Infect. 2016 Dec 21;5(12):e126–. doi: 10.1038/emi.2016.125 (PMC5180369; doi:10.1038/emi.2016.125)
Supplement: Supplementary Figure 1 [file emi2016125x1.pdf]

Number	GenBank ID	S2' (residues 880-895)	Description
1	AFS88936.1	STGS**R**SA**R**SAIEDLLF	EMC/2012 (reference)
2	AHL18090.1	......S.I.......	NRCE-HKU205
3	AIZ74439.1	....	
4	ALS20350.1	....	
5	AMO03401.1	....	
6	AHY21469.1	....	
7	AGH58717.1	....	
8	AHY22555.1	....	
9	AHY22565.1	....	
10	AKM76239.1	....	
11	AJG44080.1	....	
12	AJG44069.1	....	
13	AIZ48769.1	....	
14	AHX00731.1	....	
15	AHX00721.1	....	
16	AHX00711.1	....	
17	AHI48616.1	....	
18	AKN24812.1	....	
19	ALA49814.1	....	
20	ALA49803.1	....	
21	ALA49649.1	....	
22	AGV08438.1	....	
23	AHI48733.1	....	
24	ALR69641.1	....	
25	ALJ54461.1	....	
26	AHZ90568.1	....	
27	AJG44113.1	....	
28	AJG44102.1	....	
29	AKM76229.1	....	
30	AJG44091.1	....	
31	AJG44058.1	....	
32	AIY60578.1	....	
33	ALM26400.1	....	
34	AIY60588.1	....	
35	AIY60568.1	....	
36	AIY60558.1	....	
37	AIY60548.1	....	
38	AIY60538.1	....	

39	AIY60518.1	. . . .
40	AIY60528.1	. . . .
41	AJG44124.1	. . . .
42	AHB33326.1	. . . .
43	AIZ74417.1	. . . .
44	AIZ74405.1	. . . .
45	AIZ74450.1	. . . .
46	AIZ74433.1	. . . .
47	ALA49671.1	. . . .
48	ALA49660.1	. . . .
49	ALJ54495.1	. . . .
50	ALJ76285.1	. . . .
51	ALJ76278.1	. . . .
52	ALW82753.1	. . . .
53	ALJ54452.1	. . . .
54	ALJ54474.1	. . . .
55	ALJ76286.1	. . . .
56	ALJ76277.1	. . . .
57	ALA50067.1	. . . .
58	ALA49429.1	. . . .
59	ALA49418.1	. . . .
60	ALA49407.1	. . . .
61	ALA49396.1	. . . .
62	ALJ54486.1	. . . .
63	ALJ54484.1	. . . .
64	ALJ54481.1	. . . .
65	ALJ54450.1	. . . .
66	AIZ48760.1	. . . .
67	AHZ64057.1	. . . .
68	AID55087.1	. . . .
69	AID55085.1	. . . .
70	AID55079.1	. . . .
71	AHZ20790.1	. . . .
72	AID55084.1	. . . .
73	ALJ54472.1	. . . .
74	AID55097.1	. . . .
75	AGV08524.1	. . . .
76	AGV08492.1	. . . .
77	ALJ54446.1	. . . .

78	ALA49341.1	....
79	AID55090.1	....
80	AID55095.1	....
81	ALJ54451.1	....
82	ALJ54455.1	....
83	ALJ54490.1	....
84	ALJ54456.1	....
85	AHI48528.1	....
86	AHI48583.1	....
87	AHI48550.1	....
88	AGV08390.1	....
89	AGN52936.1	....
90	K9N5Q8.1	....
91	AGG22542.1	....
92	AFY13307.1	....
93	AHI48739.1	....
94	AHI48737.1	....
95	AHI48702.1	....
96	ALJ54496.1	....
97	ALJ54502.1	....
98	ALA49495.1	....
99	ALA49473.1	....
100	ALJ54517.1	....
101	ALK80311.1	....
102	ALK80301.1	....
103	ALK80261.1	....
104	ALK80251.1	....
105	AKN11075.1	....
106	AKN11074.1	....
107	AKL59401.1	....
108	ALJ54471.1	....
109	ALB08322.1	....
110	ALB08311.1	....
111	AMW90852.1	....
112	ALA49704.1	....
113	ALA49693.1	....
114	ALA49374.1	....
115	AKN24830.1	....
116	AID55096.1	....

117	AKN24803.1	....
118	AMW90853.1	....
119	AMW90843.1	....
120	ALW82731.1	....
121	ALW82720.1	....
122	ALW82709.1	....
123	ALW82702.1	....
124	ALW82680.1	....
125	ALW82669.1	....
126	ALW82658.1	....
127	ALW82647.1	....
128	ALW82636.1	....
129	ALT66824.1	....
130	ALT66813.1	....
131	ALT66802.1	....
132	ALK80242.1	....
133	ALJ54519.1	....
134	ALJ54515.1	....
135	ALJ54514.1	....
136	ALJ54513.1	....
137	ALJ54511.1	....
138	ALJ54510.1	....
139	ALJ54509.1	....
140	ALJ54507.1	....
141	ALJ54506.1	....
142	ALJ54505.1	....
143	ALJ54504.1	....
144	ALJ54503.1	....
145	ALJ54499.1	....
146	ALJ54498.1	....
147	ALJ54494.1	....
148	ALJ54492.1	....
149	ALJ54489.1	....
150	ALJ54487.1	....
151	ALJ54485.1	....
152	ALJ54482.1	....
153	ALJ54476.1	....
154	ALJ54473.1	....
155	ALJ54470.1	....

156	ALJ54469.1	....
157	ALJ54464.1	....
158	ALJ54462.1	....
159	ALJ54460.1	....
160	ALJ54459.1	....
161	ALJ54457.1	....
162	ALJ54454.1	....
163	ALJ54449.1	....
164	ALJ54447.1	....
165	ALJ54445.1	....
166	ALJ54444.1	....
167	ALA50056.1	....
168	ALA50023.1	....
169	ALA50012.1	....
170	ALA50001.1	....
171	ALA49990.1	....
172	ALA49979.1	....
173	ALA49935.1	....
174	ALA49924.1	....
175	ALA49913.1	....
176	ALA49891.1	....
177	ALA49880.1	....
178	ALA49869.1	....
179	ALA49858.1	....
180	ALA49847.1	....
181	ALA49825.1	....
182	ALA49440.1	....
183	AKQ21073.1	....
184	AKQ21064.1	....
185	AKQ21055.1	....
186	AKN11071.1	....
187	AKL80615.1	....
188	AKL80604.1	....
189	AKL80593.1	....
190	AKK52612.1	....
191	AKK52602.1	....
192	AKK52592.1	....
193	AKK52582.1	....
194	AKJ80137.2	....

195	AKA63509.1	....
196	AJD81451.1	....
197	AID55092.1	....
198	AID50418.1	....
199	AHY22535.1	....
200	AHY22525.1	....
201	AHI48662.1	....
202	AHI48572.1	....
203	AHI48731.1	....
204	ALJ76284.1	....
205	ALJ76283.1	....
206	ALJ76282.1	....
207	ALJ76281.1	....
208	ALJ76280.1	....
209	ALJ76279.1	....
210	ALJ54493.1	....
211	ALJ54480.1	....
212	ALJ54479.1	....
213	ALJ54478.1	....
214	ALJ54477.1	....
215	ALJ54475.1	....
216	ALA50045.1	....
217	ALA50034.1	....
218	ALA49682.1	....
219	ALA49638.1	....
220	ALA49627.1	....
221	ALA49616.1	....
222	ALA49572.1	....
223	ALA49561.1	....
224	ALA49550.1	....
225	ALA49385.1	....
226	AID55102.1	....
227	AID55086.1	....
228	AID55083.1	....
229	AID55082.1	....
230	AID55081.1	....
231	AID55080.1	....
232	AID55078.1	....
233	AID55077.1	....

234	AID55076.1	....
235	AID55075.1	....
236	AID55074.1	....
237	AID55072.1	....
238	AID55071.1	....
239	AID55070.1	....
240	AID55069.1	....
241	AID55068.1	....
242	AID55067.1	....
243	AID55066.1	....
244	AMW90854.1	....
245	AMW90851.1	....
246	AMW90850.1	....
247	AMW90849.1	....
248	AMW90848.1	....
249	AMW90847.1	....
250	AMW90846.1	....
251	AMW90845.1	....
252	AKN24821.1	....
253	AKN24794.1	....
254	AKN24785.1	....
255	AKN24776.1	....
256	AKN24767.1	....
257	AKN24758.1	....
258	AKN24749.1	....
259	AID55103.1	....
260	AID55101.1	....
261	AID55100.1	....
262	AID55098.1	....
263	AID55094.1	....
264	AID55093.1	....
265	AID55091.1	....
266	AID55089.1	....
267	AID55073.1	....
268	AHZ58501.1	....
269	ALA49792.1	....
270	ALA49781.1	....
271	ALA49770.1	....
272	ALA49759.1	....

273	ALA49748.1	....
274	ALA49737.1	....
275	ALA49726.1	....
276	ALA49715.1	....
277	ALA49605.1	....
278	ALA49594.1	....
279	ALA49583.1	....
280	ALA49539.1	....
281	ALA49528.1	....
282	ALA49517.1	....
283	ALA49506.1	....
284	ALA49484.1	....
285	ALA49462.1	....
286	YP9047204	....
287	AKN11076.1	....
288	AHN10812.1	....
289	AHI48605.1	....
290	AHI48594.1	....
291	AHE78108.1	....
292	AHE78097.1	....
293	AMW90844.1	....
294	AID55088.1	....
295	ALW82742.1	....
296	ALW82691.1	....
297	ALJ54518.1	....
298	ALK80291.1	....
299	ALK80281.1	....
300	ALK80271.1	....
301	ALK80232.1	....
302	ALK80222.1	....
303	ALK80212.1	....
304	ALK80202.1	....
305	ALK80192.1	....
306	ALB08300.1	....
307	ALB08289.1	....
308	ALB08278.1	....
309	ALB08267.1	....
310	AKN11073.1	....
311	AKN11072.1	....

312	ALJ54520.1	....
313	AGV08584.1	....
314	ALJ54501.1	....
315	ALJ54516.1	....
316	ALJ54512.1	....
317	ALJ54508.1	....
318	ALJ54500.1	....
319	ALJ54497.1	....
320	ALJ54488.1	....
321	ALJ54491.1	....
322	ALJ54483.1	....
323	ALJ54458.1	....
324	ALJ54468.1	....
325	AKO69636.1	....
326	AKO69634.1	....
327	ALJ54467.1	....
328	ALJ54466.1	....
329	ALJ54465.1	....
330	ALJ54448.1	....
331	ALJ54463.1	....
332	ALA49352.1	....
333	ALJ54453.1	....
334	ALD51904.1	....
335	ALB08257.1	....
336	ALB08246.1	....
337	ALA49968.1	....
338	ALA49957.1	....
339	ALA49946.1	....
340	ALA49902.1	....
341	ALA49836.1	....
342	ALA49451.1	....
343	ALA49363.1	....
344	AKO69635.1	....
345	AHI48682.1	....
346	AHI48626.1	....
347	AHI48561.1	....
348	AHI48539.1	....
349	AGV08455.1	....
350	AJD81440.1	....

351	AID55099.1	....
352	AHY22545.1	....
353	AHX71946.1	....
354	AHC74098.1	....
355	AHC74088.1	....
356	AGV08408.1	....
357	AGV08379.1	....
358	AGV08467.1	....
359	AHI48692.1	....
360	ALJ54443.1	....
361	ALJ54442.1	....
362	ALJ54441.1	....
363	AKI29284.1	....
364	AKI29275.1	....
365	AKI29265.1	....
366	AKI29255.1	....
367	ALJ54521.1	....
368	ALT66880.1	....
369	ALT66870.1	....
370	AHI48672.1	....
371	AHI48727.1	....
372	AHI48711.1	....
373	AHI48652.1	....
374	AHI48517.1	....
375	AGV08573.1	....
376	AGV08558.1	....
377	AGV08546.1	....
378	AGV08535.1	....
379	AGV08505.1	....
380	AGV08480.1	....
381	AGV08444.1	....
382	AGV08426.1	....
383	AGN70973.1	....
384	AGN70962.1	....
385	AGN70951.1	....
386	AGN70929.1	....

Supplementary Figure S1 Protein sequence alignment of the spike S2' site of 386 human and camel MERS-CoV. The S2' spike cleavage site of human and camel MERS-CoV were aligned using the ClustalW alignment method in Geneious software. Dots indicate identical residues than the ones in reference EMC/2012 S2' sequence.
