# Supplementary figures and images for: A camel-derived MERS-CoV with a variant spike protein cleavage site and distinct fusion activation properties
Source: Emerg Microbes Infect. 2016 Dec 21;5(12):e126–. doi: 10.1038/emi.2016.125 (PMC5180369; doi:10.1038/emi.2016.125)

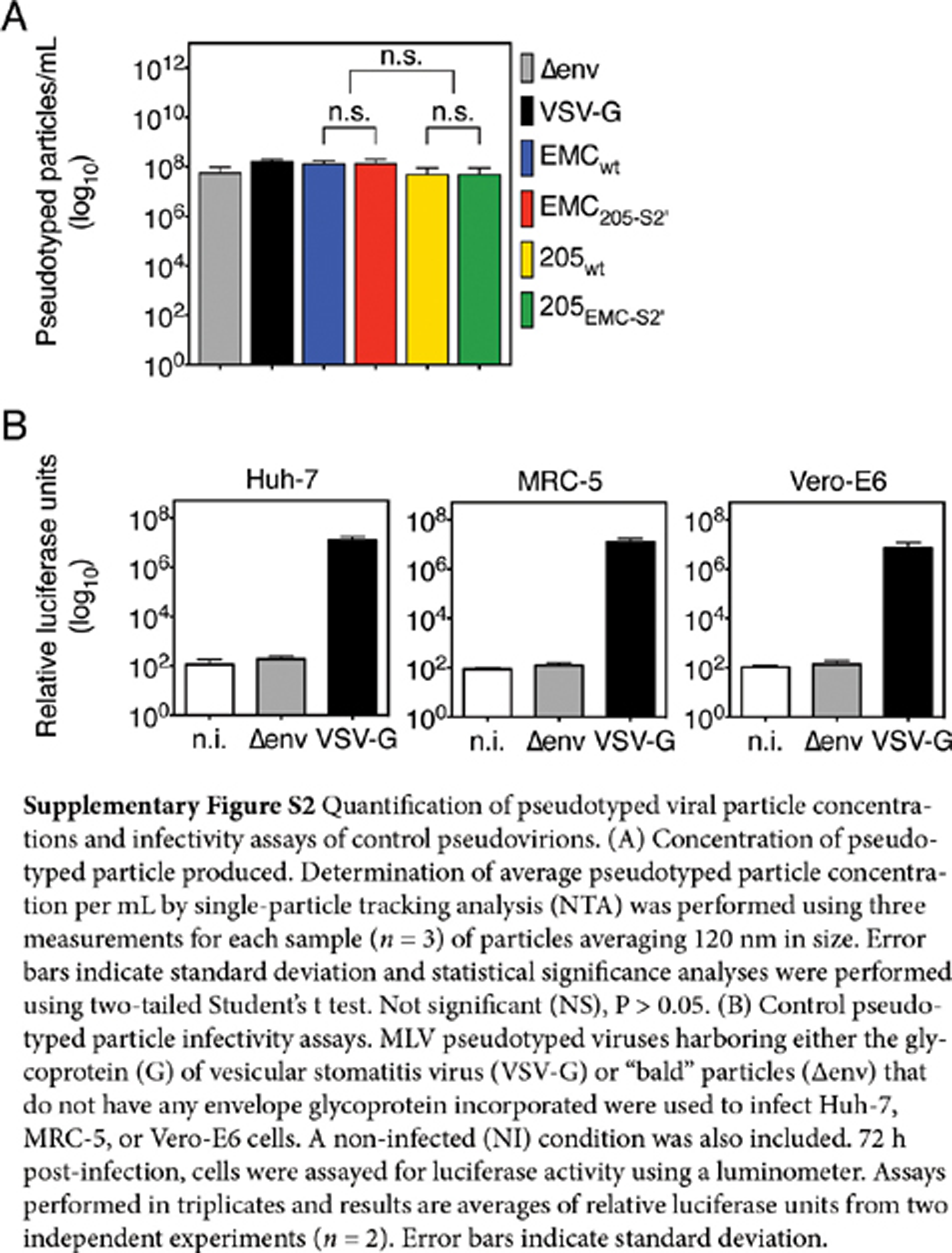

Supplement: Supplementary Figure 2 [file emi2016125x2.tif]
